# Development of Liposomal Formulations for 1,4-bis-L/L Methionine-Conjugated Mitoxantrone–Amino Acid Conjugates to Improve Pharmacokinetics and Therapeutic Efficacy

**DOI:** 10.3390/pharmaceutics17091226

**Published:** 2025-09-21

**Authors:** Ting-Lun Yang, Tsai-Kun Li, Chin-Tin Chen

**Affiliations:** 1 Department of Pharmacy, College of Pharmacy and Science, Chia Nan University of Pharmacy and Science, Tainan 717301, Taiwan; tinglun0724@mail.cnu.edu.tw; 2 Department of Biochemical Science and Technology, National Taiwan University, Taipei 106319, Taiwan; 3 Graduate Institute of Microbiology, National Taiwan University College of Medicine, Taipei 10051, Taiwan; tsaikunli@ntu.edu.tw; 4 Development Center for Biotechnology, Taipei 115, Taiwan; 5 Department and Graduate Institute of Biochemistry, National Defense Medical Center, Taipei 11490, Taiwan

**Keywords:** liposome, topoisomerase II, 1,4-bis-L/L methionine-conjugated mitoxantrone–amino acid conjugates, remote loading, ammonium sulfate

## Abstract

**Background:** 1,4-bis-L/L methionine–conjugated mitoxantrone–amino acid conjugate (L/LMet-MAC) inhibits topoisomerase IIα and enhances tumor cytotoxicity, but its short half-life limits therapeutic application. **Objective:** To improve the pharmacokinetics and antitumor efficacy of L/LMet-MAC through liposomal encapsulation. **Methods:** PEGylated DSPC liposomes containing EPG or prepared via the ammonium sulfate gradient method were employed to encapsulate L/LMet-MAC. Encapsulation efficiency, drug-to-lipid ratio, and serum stability were assessed. Pharmacokinetics, antitumor efficacy, and systemic safety were further evaluated in vivo. **Results:** L/LMet-MAC encapsulated in PEGylated DSPC liposomes containing EPG or prepared using the ammonium sulfate gradient method has high encapsulation efficiency. Further studies show that PEGylated DSPC liposomes prepared with the ammonium sulfate gradient approach display an efficient D/L ratio and serum stability as well as improved pharmacokinetics and enhanced antitumor efficacy while mitigating the side effects of L/LMet-MAC. **Conclusions:** PEGylated DSPC liposomes prepared using an ammonium sulfate gradient showed favorable performance for delivering L/LMet-MAC.

## 1. Introduction

Topoisomerase II is a crucial enzyme in DNA replication and mitosis, and modulates DNA supercoiling, knotting, and catenation. There are two isoforms in vertebrates, topoisomerase IIα and topoisomerase IIβ, which have similar structures but different biological functions [1]. Topoisomerase IIβ expresses consistently in cells and is involved in transcriptional regulation and tissue differentiation [2,3]. In contrast, topoisomerase IIα is crucial for cell proliferation, and is involved in the process of chromosomal condensation and segregation during mitosis. The expression of topoisomerase IIα is cell-cycle-regulated and crucial for DNA replication. Overexpression of topoisomerase IIα has been found in tumor tissues and linked to cancer progression, making it a molecular target for anticancer drugs [4,5]. Topoisomerase II poisons, such as anthracycline analogues, can stabilize topoisomerase II–DNA complexes and act as physical barriers to prevent the unraveling of DNA supercoiled structures [6]. However, these drugs can bind to not only topoisomerase IIα but also topoisomerase IIβ, which has relatively high expression in myocardial cells, resulting in anthracycline-related cardiotoxicity [7]. For example, doxorubicin is an anthracycline chemotherapeutic drug which can intercalate into DNA duplex, and interfere with the normal function of topoisomerase II [8]. However, doxorubicin-induced cardiomyopathy causes an increased risk of patient mortality, limiting its wide clinical application [9]. One strategy to reduce cardiotoxicity is to develop novel anthracycline analogues, which specifically interact with topoisomerase IIα. Mitoxantrone belongs to the anthracenedione class of compounds, with a pharmacological mechanism similar to that of doxorubicin [10]. Although the cardiotoxicity induced by mitoxantrone is significantly less severe than that of doxorubicin, the increasing risk with cumulative dose still limits its clinical use [11].

1,4-bis-L/L methionine-conjugated mitoxantrone–amino acid conjugate (L/LMet-MAC) is an anthracenedione compound specifically designed to target topoisomerase IIα [12]. This synthetic antineoplastic agent has demonstrated superior tumor suppressibility in vitro compared to that of mitoxantrone and a higher maximum tolerated dose (MTD) in vivo, suggesting that it can be administered at higher doses with fewer adverse effects [12]. This provides a broader therapeutic window, which is highly desirable in oncology, where balancing efficacy and toxicity is critical. Additionally, the characteristic of methionine conjugation in L/LMet-MAC renders it a poor substrate for ABC transporters, which minimizes the risk of drug resistance, making it a promising candidate for long-term cancer treatment [12]. However, in that in vivo animal study, L/LMet-MAC was administrated intraperitoneally every 3.5 days [12], which is a relatively frequent dosing schedule compared to other more potent anticancer agents. Such a high dosing regimen could create issues in clinical settings, such as reducing patient compliance and increasing the treatment burden.

Liposome is a well-recognized therapeutic nanocarrier for its ability to extend the half-life of active pharmaceutical ingredients (APIs) by protecting them from physiological degradation, and to alter their biodistribution, which can ultimately deliver APIs more efficiently to the target tissue through passive or active targeting, thus reducing toxicity to other organs [13,14]. Additionally, PEGylation of liposomes can not only stabilize them to prevent premature drug release but also prolong their circulation in the bloodstream [15,16], therefore reducing the dosing frequency and enhancing the overall therapeutic efficacy [15,17]. In this regard, encapsulating L/LMet-MAC within a liposomal delivery system is a compelling approach to increase its circulation time, improve drug stability, and optimize its therapeutic potential in vivo, ultimately leading to better therapeutic outcomes.

In this study, three types of L/LMet-MAC-loaded PEGylated DSPC liposomes were developed using the thin-film hydration method. We found that PEGylated DSPC liposome prepared in the presence of Egg L-α-phosphatidylglycerol (EPG) or via the ammonium sulfate gradient approach has high encapsulation efficiency (EE %). Moreover, the physiochemical properties, drug-to-lipid (D/L) ratio, and stability were characterized in these liposomal formulations. Finally, the pharmacokinetic behaviors and therapeutic efficacy of the selected L/LMet-MAC-loaded liposomes were further investigated in BALB/c mice.

## 2. Materials and Methods

### 2.1. Materials

1,2-distearoyl-*sn*-glycero-3-phosphocholine (DSPC), 1,2-distearoyl-sn-glycero-3-phosphoethanolamine-N-[amino(polyethylene glycol)-2000] (ammonium salt) (DSPE-PEG 2000), and Egg L-α-phosphatidylglycerol (sodium salt) (EPG) were purchased from Avanti Polar Lipids, Inc. (Alabaster, AL, USA). Fetal bovine serum (FBS) was purchased from Biological Industries (Kibbutz Beit Haemek, Israel). Thiazolyl blue tetrazolium bromide was purchased from Alfa Aesar (Ward Hill, MA, USA). Chloroform was purchased from J.T. Baker Chemicals (Suwon, Gyeonggi-do, Republic of Korea). Ethylenediaminetetraacetic acid (EDTA), phosphorus standard solution, ammonium sulfate, ammonium molybdate, ascorbic acid, cholesterol, dimethyl sulfoxide (DMSO), hydrogen peroxide, hydrochloric acid (HCl), sulfuric acid, phosphate-buffered saline (PBS), and Roswell Park Memorial Institute 1640 (RPMI 1640) medium were purchased from Sigma-Aldrich (St. Louis, MO, USA). Methyl alcohol, anhydrous was purchased from Macron Fine Chemicals (Radnor, PA, USA). Sephadex G-50 and Sepharose CL-4B were purchased from GE Healthcare Life Science (Uppsala, Sweden). Ethyl alcohol (EtOH), 95%, was purchased from Taiwan Sugar Corporation (Tainan, Taiwan). Polycarbonate 0.1 µm pore size membrane was purchased from Whatman PLC (Maidstone, Kent, UK). 1,4-bis-L/l-methionine-conjugated mitoxantrone–amino acid conjugates (L/LMet-MAC) were provided by Dr. Tsai-Kun Li, Graduate Institute of Microbiology, National Taiwan University College of Medicine, Taipei, Taiwan.

### 2.2. Preparation of Different Types of PEGylated Liposomes Encapsulated with L/LMet-MAC

Three types of L/LMet-MAC-loaded PEGylated liposomes were prepared using the thin-film hydration method with their respective lipid compositions and drug loading methods as described below. The first one was a conventional PEGylated DSPC liposome with a lipid composition including DSPC, cholesterol, and DSPE-PEG for increased stability and longer circulation. The compositions of DSPC/EPG PEGylated liposome included DSPC, cholesterol, DSPE-PEG, and different ratios of EPG. Briefly, the lipid mixtures were homogeneously dissolved in chloroform and evaporated in a rotary evaporator (BÜCHI Rotavapor R-124, Flawil, Switzerland) at 65 °C, 55 rpm until the formation of a thin lipid film. This film was subsequently rehydrated with 0.9% NaCl containing 0.5 or 1.0 mg/mL L/LMet-MAC. Then, the lipid suspension was frozen and thawed in liquid nitrogen and a 65 °C water bath, followed by sonication for 30 min at 65 °C. The resultant mixtures were extruded 10 times at 65 °C through 0.1 µm pore size polycarbonate membranes. The lipid composition of the gradient PEGylated DSPC liposome was the same as in the conventional liposome, except loading of L/LMet-MAC was performed via ammonium sulfate gradient. Briefly, a lipid mixture of DSPC/DSPE-PEG 2000/cholesterol (10:0.2:5 µmole) was dissolved in chloroform and evaporated in a rotary evaporator. The lipid film was then rehydrated with 250 mM ammonium sulfate without loading drugs. After size extrusion and gel filtration, 0.5 or 1.0 mg/mL L/LMet-MAC dissolved in 0.9% NaCl was added to the lipid suspension and incubated in a 65 °C water bath for 30 min.

The non-encapsulated drugs in the three types of PEGylated liposomes were separated by passing through Sephadex G-50 gel filtration chromatography. The final dispersion containing PEGylated liposomal L/LMet-MAC was suspended in 0.9% NaCl and stored at 4 °C for further study.

### 2.3. Characterization of PEGylated Liposomal L/LMet-MAC

The particle size was measured with the dynamic light scattering method using a particle sizer (SZ-100, HORIBA, Kyoto, Japan). Cryogenic transmission electron microscopy (cryo-TEM, Tecnai F20, Philips, Eindhoven, The Netherlands) was utilized for observing the morphology of liposomal L/LMet-MAC. The amount of L/LMet-MAC encapsulated in the liposomes was determined by disrupting the phospholipid bilayer with methyl alcohol to release the drug which was measured with a UV–Visible Spectrophotometer DU8000 (Beckman Coulter, CA, USA) at a wavelength of 672 nm. The amount of phospholipid of the liposomes was determined according to Bartlett assay [18]. Briefly, the phospholipid was first digested by sulfuric acid and then oxidized to inorganic phosphate by hydrogen peroxide at 200 °C. The inorganic phosphate mixture was then combined with ammonium molybdate to transform into phosphomolybdate, followed by reduction through ascorbic acid at 100 °C. The final blue compound was measured colorimetrically at 830 nm for the quantitative assessment of the phospholipids. The equation of encapsulation efficiency (E.E.) and lipid recovery is as follows:

(1) $$E.E.\left(\%\right)=\frac{\mathrm{amount}\;\mathrm{of}\;L/\mathrm{LMet}-\mathrm{MAC}\;\mathrm{in}\;\mathrm{liposomes}\;\mathrm{after}\;\mathrm{gel}\;\mathrm{filtration}}{\mathrm{theoretical}\;\mathrm{amount}\;\mathrm{of}\;L/\mathrm{LMet}-\mathrm{MAC}\;\mathrm{initially}\;\mathrm{added}\;\mathrm{during}\;\mathrm{liposome}\;\mathrm{preparation}}\;\times \;100\%$$

(2) $$\mathrm{Lipid}\;\mathrm{recovery}\left(\%\right)=\frac{\mathrm{amount}\;\mathrm{of}\;\mathrm{phosphate}\;\mathrm{in}\;\mathrm{liposomes}\;\mathrm{after}\;\mathrm{gel}\;\mathrm{filtration}}{\mathrm{theoretical}\;\mathrm{amount}\;\mathrm{of}\;\mathrm{phosphate}\;\mathrm{initially}\;\mathrm{added}\;\mathrm{for}\;\mathrm{liposome}\;\mathrm{preparation}}\;\times \;100\%$$

### 2.4. Stability Assay of PEGylated Liposomal L/LMet-MAC

For the long-term stability test, aliquots of different types of liposomal L/LMet-MAC were placed into an Eppendorf tube in 0.9% NaCl and kept in the dark at 4 °C or 37 °C. At different time points, the liposomal drug from the stored Eppendorf tube was passed through a Sepharose CL-4B packed column to collect liposomes encapsulated with L/LMet-MAC. The percentage of encapsulated L/LMet-MAC in the liposomal formula was used to assess the long-term stability of liposomes.

Spin column filtration assay was performed to investigate the serum stability of different types of liposomal L/LMet-MAC according to the study by Soundararajan, A. et al., with some modifications [19]. Briefly, liposomal L/LMet-MAC was incubated in a mixture containing 20% fetal bovine serum (FBS) at 37 °C for various time intervals. The mixture was prepared by diluting 100% FBS with phosphate-buffered saline (PBS), at pH 7.4. The final concentration of liposomal L/LMet-MAC in the FBS/PBS mixture was 3.5 μg/mL. At the desired time points, 50 μL of liposomal L/LMet-MAC in serum solution was added to the equilibrated Sepharose CL-4B spin column and centrifuged at 1000 rpm for 1 min to collect the first fraction. Then, 50 μL of 0.9% NaCl (pH 7.4) was added to the spin column and centrifuged at 1000 rpm for 1 min to collect the second fraction. This elution process was repeated 6 times and each fraction was collected after centrifugation for further analysis. L/LMet-MAC in each collected fraction was treated with methyl alcohol to extract it from the liposome. The extracted L/LMet-MAC was quantified by measuring the fluorescence intensity at a wavelength of excitation/emission of 668/672 nm in a spectrofluorometer (FluoroMax1-4, Horiba Jobin Yvon, NJ, USA). The equation of the residual percentage of L/LMet-MAC in liposomes was calculated as follows:

(3) $$\mathrm{Residual}\;\mathrm{pencentage}(\%)=\frac{\mathrm{amount}\;\mathrm{of}\;L/\mathrm{LMet}-\mathrm{MAC}\;\mathrm{in}\;\mathrm{liposomes}\;\mathrm{after}\;\mathrm{spin}\;\mathrm{column}\;\mathrm{filtration}}{\mathrm{amount}\;\mathrm{of}\;L/\mathrm{LMet}-\mathrm{MAC}\;\mathrm{in}\;\mathrm{liposomes}}\;\times \;100\%$$

### 2.5. Analysis of Cell Viability with MTT Assay

Murine colon-26 (C26) cells obtained from the Bioresource Collection and Research Center (BCRC) (Hsinchu, Taiwan) were used in the in vitro and in vivo experiments. C26 cells were grown in RPMI 1640 medium containing 10% FBS at 37 °C in a humidified atmosphere of 5% CO_2_. The MTT assay was performed according to the study by Angius et al., with modification [20]. Briefly, C26 cells seeded in the 96-well culture plate were incubated with different concentrations of the liposomal drugs for 24 h. The non-uptake drugs were removed and washed with PBS three times. Then, the cells were cultured in RPMI 1640 medium containing 10% FBS for another 24 h. After removing the medium, MTT solution was added and incubated in the dark for 2 h. The obtained blue/purple crystalline product, formazan, was then dissolved in DMSO. The amount of formazan was determined using a Microplate Reader (Anthos-Elisa-reader 2001, Labtec, Heerhugowaard, The Netherlands) at a wavelength of 570 nm. The equation of cell viability (%) is as follows:

(4) $$\mathrm{Cell}\;\mathrm{viability}\left(\%\right)=\frac{\mathrm{mean}\;\mathrm{absorbance}\;\mathrm{of}\;\mathrm{treated}\;\mathrm{cells}}{\mathrm{mean}\;\mathrm{absorbance}\;\mathrm{of}\;\mathrm{control}\;\mathrm{cells}}\;\times \;100\%$$

### 2.6. Animal Experiments in Mice

#### 2.6.1. Pharmacokinetic Studies in Healthy Female BALB/c Mice

Eight-week-old healthy BALB/c mice were injected intravenously with a single dose of 5 mg/kg liposomal L/LMet-MAC. At the indicated time point, 50 μL blood samples was collected from the orbital sinus using a heparinized capillary tube and placed in 200 μL PBS containing 0.5 mM EDTA. After removing the blood cells, the collected serum was mixed with acidified alcohol (95% EtOH + 0.6 N HCl) at a ratio of 1:5 (*v*/*v*) and kept at 4 °C for 24 h for extracting drugs and precipitating proteins. The precipitated proteins were removed using centrifugation at 12,000 rpm for 10 min. The amount of L/LMet-MAC in the resultant supernatant was quantified as described above in a spectrofluorometer. The pharmacokinetic parameters were assessed using the non-compartmental IV bolus model in PKsolver [21].

#### 2.6.2. In Vivo Therapeutic Effect in C26 Bearing Mice

The therapeutic efficacy in vivo was examined in the C26-bearing BALB/c mice. The protocols were approved by the Institutional Animal Care and Use Committee (IACUC) of the National Taiwan University (IACUC Approval No.: NTU-110-EL-00003). Briefly, 2 × 10^5^ C26 cells were subcutaneously injected into the back of 8-week-old female BALB/c mice, which were purchased from the National Laboratory Animal Center (Taipei, Taiwan). When the tumor size grew to 100 mm^3^, 2 mg/kg of free-form or liposomal L/LMet-MAC or 0.9% normal saline was injected intravenously. Mice in the respective groups were given an injection of the drug every 7 days for three consecutive weeks. Tumor size was measured every 3 days after the first drug administration. The end point of this therapeutic study was when the tumor size exceeded 2500 mm^3^. The toxic effect was defined as a 20% reduction in original body weight. Tumor volume was calculated using the following equation:

(5) $$\mathrm{Tumor}\;\mathrm{volume}=\frac{a^{2}\times b}{2}$$

where a is the smallest and b is the largest perpendicular diameter.

## 3. Results and Discussion

### 3.1. Development and Charcteristerization of Different Types of PEGylated Liposome-Encapsulated L/LMet-MAC

As shown in Table 1, three types of PEGylated liposomes were prepared to encapsulate L/LMet-MAC using the thin-film hydration method. As reported, DSPC-based liposomes possess higher stability in vitro and longer drug half-life in vivo due to the higher phase transition temperature compared to HSPC [22,23]. In this regard, we first prepared conventional PEGylated DSPC liposome with a lipid composition consisting of DSPC, cholesterol, and DSPE-PEG 2000 (molar ratio of 10:5:0.2) to encapsulate L/LMet-MAC. The drug encapsulation efficiency of this conventional DSPC liposome was only approximately 20% (Table 1). Due to the positive charge of L/LMet-MAC under neutral conditions, we further included egg phosphatidylglycerol (EPG), an anionic phospholipid, into the liposome to increase the encapsulation efficiency. As shown in Table 1, the encapsulation efficiency of DSPC/EPG liposomes significantly increased to 77% at a 1:1 molar ratio of DSPC/EPG under the drug loading dose of 0.5 mg/mL. Although the encapsulation efficiency of DSPC/EPG liposome did not increase when the loading dose of L/LMet-MAC increased to 1.0 mg/mL, there was a twofold increase in the drug-to-lipid ratio (D/L value).

**Figure 1:**
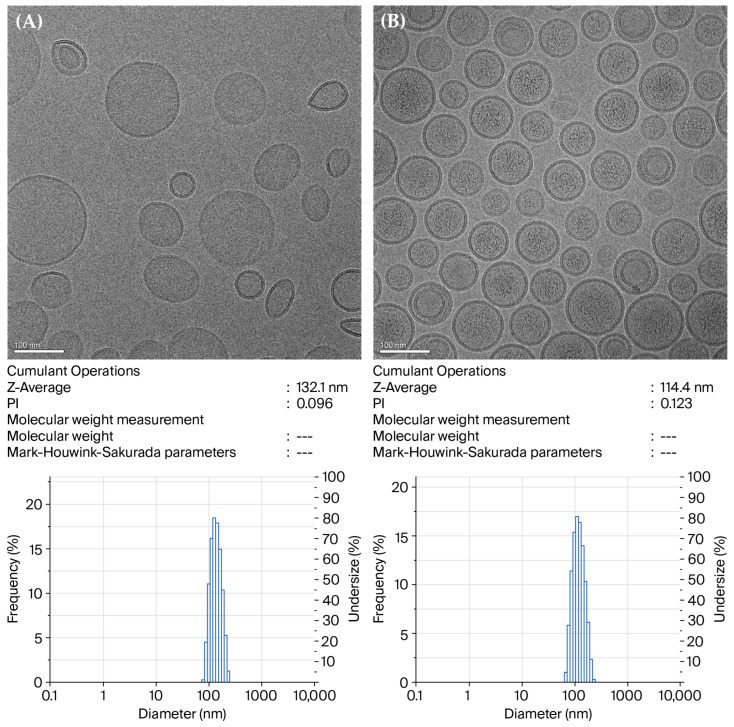
Representative Cryo-EM morphology of DSPC/EPG liposomal L/LMet-MAC (**A**) and gradient DSPC liposomal L/LMet-MAC made with ammonium sulfate (**B**). The molar ratio of DSPC, EPG, cholesterol, and DSPE-PEG 2000 of DSPC/EPG-liposome and ammonium sulfate gradient liposome was 5:5:5:0.2 and 10:0:5:0.2, respectively. The loading dose of L/LMet-MAC was 1.0 mg/mL for both formulations. The lower panels show particle size distributions captured directly from the original output of the particle sizer (SZ-100, HORIBA, Kyoto, Japan).

Remote loading has been shown to be an efficient method for encapsulating amphipathic drugs into liposomes with high encapsulated efficiency and long-term stability, such as the use of ammonium sulfate gradient for loading doxorubicin [24]. Due to L/LMet-MAC having a similar structure to doxorubicin [12], the ammonium sulfate gradient method was also used for the development of liposomal L/LMet-MAC. Table 1 shows that the ammonium sulfate gradient approach used to load L/LMet-MAC into PEGylated DSPC liposomes exhibited a significantly higher encapsulation efficiency than those prepared using the conventional passive loading method. In fact, the higher encapsulated efficiency correlates with the co-precipitation of L/LMet-MAC with sulfate ions in the aqueous core of liposome (Figure 1B), as found in the liposomal doxorubicin prepared using the ammonium sulfate method [25]. To summarize, a significantly higher L/LMet-MAC encapsulation efficiency could be found in the PEGylated DSPC liposome either containing DSPC and EPG at a 1:1 molar ratio or made using the ammonium sulfate gradient method.

The particle size of the conventional DSPC liposomal L/LMet-MAC is around 104 nm (Table 1). In contrast, the DSPC/EPG liposomes have a larger particle size of around 115 nm and 132 nm at the drug loading dose of 0.5 mg/mL and 1.0 mg/mL, respectively. Compared to the conventional DSPC liposome, the increased particle size of DSPC/EPG liposomes might relate to the increased amounts of L/LMet-MAC, which interacts with the EPG lipids of the liposomal membrane during the synthesis process of DSPC/EPG liposome. In contrast, transmembrane ammonium sulfate gradient was used to load L/LMet-MAC into gradient DSPC liposome. During the remote loading process, the increased amount of L/LMet-MAC was further concentrated in the aqueous core of the gradient DSPC liposome. This might explain why there was no significant difference in the particle size of gradient DSPC liposome when the loading dose increased from 0.5 to 1.0 mg/mL.

Drug-to-lipid ratio (D/L value) can be used to explore the effectiveness of the preparation method, relevant pharmacokinetics, and therapeutic efficacy of liposomes [26]. In an in vivo study, Johnston, MJ et al. reported that the drug half-life could be extended at least 6 times when the D/L value was 0.05~0.39 (wt/wt) [27]. Another study demonstrated that the preferable therapeutic effects of the D/L value were in the range of 0.025~0.1 (wt/wt) but did not exceed 0.6 (wt/wt) [28]. The D/L values of DSPC/EPG liposome and gradient DSPC liposome were approximately 0.056 and 0.074 at a 0.5 mg/mL loading dose, respectively (Table 1). Furthermore, their D/L ratios increased to above 0.1 as the loading dose increased to 1.0 mg/mL, implying the drug efficacy could be significantly improved in these two liposomal formulations. Therefore, the loading dose of DSPC/EPG liposome and gradient DSPC liposome was fixed at 1.0 mg/mL for the following investigations of stability assay, in vitro cell viability test, and in vivo animal studies.

### 3.2. Storage Stability Test of DSPC/EPG Liposome and Gradient DSPC Liposome with 1.0 mg/mL L/LMet-MAC Loading Dose

#### 3.2.1. Long-Term Storage Stability at Different Temperatures

**Figure 2:**
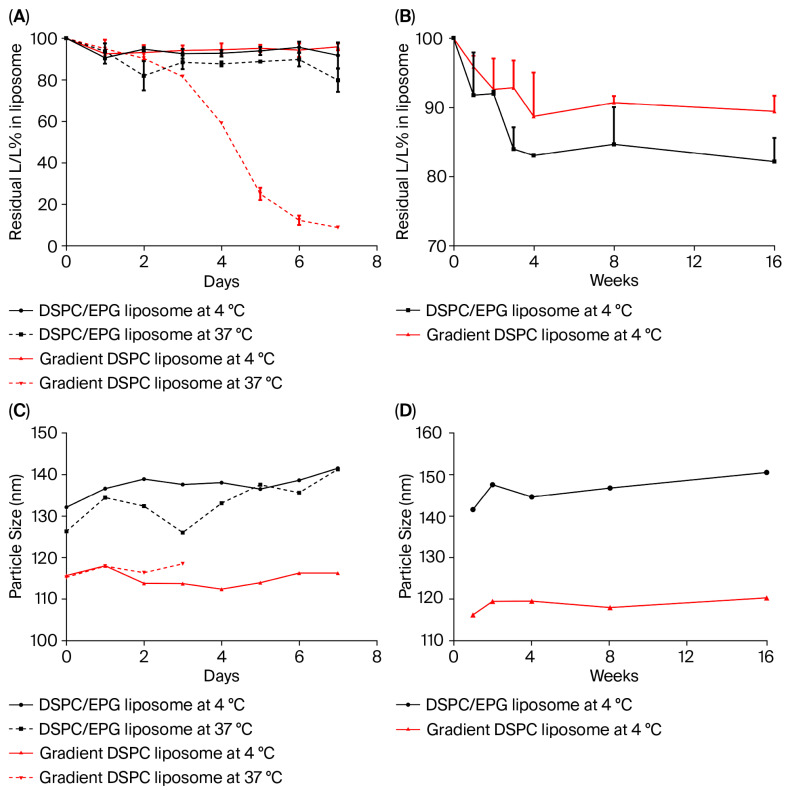
The storage stability and particle size of DSPC/EPG liposome and gradient DSPC liposome in 0.9% NaCl at 4 °C and 37 °C for different periods of time. At the indicated time point, the residual percentage of L/LMet-MAC (**A**,**B**) and particle sizes (**C**,**D**) in liposomes were analyzed during storage of 7 days and 16 weeks. Each point represents the mean ± standard deviation obtained from three independent experiments.

To investigate the storage stability of DSPC/EPG liposome and gradient DSPC liposome, liposomal L/LMet-MAC was stored at 4 °C or 37 °C for different periods of time until further analysis. As shown in Figure 2A, the leakage of L/LMet-MAC from DSPC/EPG liposome and gradient DSPC liposome was less than 10% after 7 days of storage at 4 °C. In fact, we found that the drug leakage was less than 20% for 16 weeks of storage (Figure 2B) and the particle size of both liposomes remains constant (Figure 2D). In contrast, the drug leakage from the gradient DSPC liposome dramatically increased after 3 days of storage in 0.9% NaCl at 37 °C (Figure 2A). This phenomenon might relate to the hydrolysis of amide bonds in L/LMet-MAC at higher temperature, which increases the lipophilicity and permeability of the drug. The amide hydrolysis might interfere with the co-precipitation of L/LMet-MAC with sulfate ions, leading to the drug escaping through the lipid bilayer of liposome. In addition, higher temperature might also accelerate the phospholipid oxidation, which facilitates phospholipid flip-flop in liposomes [29], thereby resulting in undetectable particle sizes of gradient DSPC liposome after 3 days of storage at 37 °C (Figure 2C). However, compared to gradient DSPC liposome, DSPC/EPG liposomes have better long-term stability in 0.9% NaCl at 37 °C (Figure 2A). This might relate to the two pairs of quaternary ammonium cation in the L/LMet-MAC [12], thus enabling the drug hydrolysate to be trapped by EPG through electrostatic attraction.

#### 3.2.2. Liposome Stability in Serum

**Figure 3:**
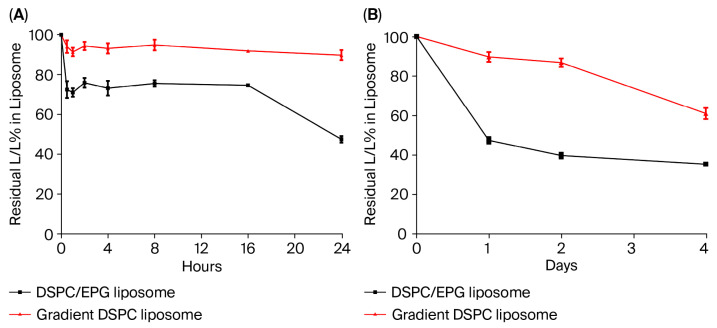
The serum stability of DSPC/EPG liposome and gradient DSPC liposome with loading dose of 1.0 mg/mL L/LMet-MAC. Liposomes were incubated in a mixture of 20% FBS and 80% phosphate buffer at 37 °C for 24 h (**A**) and 4 days (**B**). At the indicated time point, drug retention in the liposome was analyzed. Each point represents the mean ± standard deviation obtained from three independent experiments.

To examine the physiological stability of the constructed PEGylated liposomal L/LMet-MAC, a mixture of 20% fetal bovine serum (FBS) and 80% phosphate-buffered saline (PBS) was used to mimic the in vivo serum environment. The prepared liposomes were incubated in the FBS/PBS mixture at 37 °C for different periods of time and L/LMet-MAC retained in the liposome was analyzed. As shown in Figure 3A, we found that the leakage percentage of L/LMet-MAC from gradient DSPC liposome was approximately 40% after 4 days of incubation in the FBS/PBS mixture. This drug release profile is similar to that of long-term storage in 0.9% NaCl (Figure 2A). In contrast, the leakage of L/LMet-MAC from DSPC/EPG liposome was about 30% after incubation for 1 h. Then, the second leakage occurred after 16–24 h of incubation. Only 40% of L/LMet-MAC was retained in the DSPC/EPG liposome after 4 days of storage in the FBS/PBS mixture at 37 °C (Figure 3B). The serum stability of DSPC/EPG liposome is obviously different from its long-term storage stability in 0.9% NaCl, which has over 80% drug retention after 4 days of storage (Figure 2A). The stability difference of DSPC/EPG liposomal L/LMet-MAC in 0.9% NaCl and the FBS/PBS mixture could be expected. The long-term storage was examined in 0.9% NaCl, which is a simple, protein-free environment that minimally perturbs the membrane integrity of DSPC/EPG liposome. However, the FBS/PBS mixture contains proteins (e.g., albumin), lipids, bilirubin, and divalent cations such as Ca^2+^ and Mg^2+^ [30]. It has been shown that Ca^2+^ and Mg^2+^ can interact with anionic phospholipids (e.g., PG), bridging adjacent headgroups, reducing hydration, and promoting localized phase defects, thereby facilitating transient pore formation and accelerating drug leakage in anionic liposomes [31]. A study by Hirano A et al. also reported that negatively charged liposome could be disrupted by nanoscale protein [32]. In this regard, serum components might interact with DSPC/EPG liposome, thereby significantly enhancing the L/LMet-MAC leakage from the DSPC/EPG liposome. The other factor might relate to the formation of hydrolyzed L/LMet-MAC under 37 °C storage conditions. The hydrolyzed L/LMet-MAC might decrease its electrostatic interaction with EPG, resulting in its release from the DSPC/EPG liposome. These compositional and mechanistic factors explain the apparent stability discrepancy of DSPC/EPG liposomes incubated in 0.9% NaCl and the FBS/PBS mixture at 37 °C.

### 3.3. In Vitro Cell Viability Assessed Using MTT Assay

**Figure 4:**
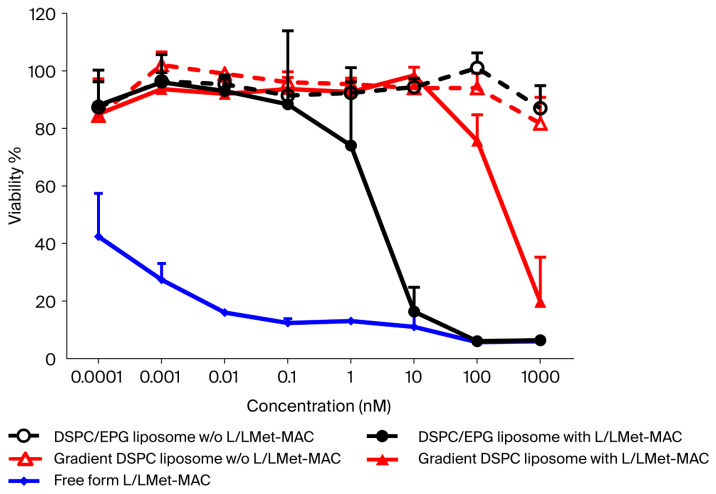
The cytotoxicities of free-form and liposomal L/LMet-MAC. Mouse C26 cells were incubated with different concentrations of drug for 24 h and cell viability was assessed with MTT assay. Data are presented as percent of control mean ± standard deviation from three independent experiments.

To compare the cytotoxicity of DSPC/EPG liposome and gradient DSPC liposome, an MTT assay was performed to examine cellular viability. As expected, free-form L/LMet-MAC significantly shows cellular killing with an IC_50_ value of less than 0.0001 nM (Figure 4). In contrast, the IC_50_ values of DSPC/EPG liposome and gradient DSPC liposome are about 5 nM and 400 nM, respectively. There is no significant cytotoxicity in both DSPC/EPG liposomes and gradient DSPC liposomes without loading L/LMet-MAC, implying that the toxicity is induced by the drug within the liposomes rather than by the liposomes themselves. In fact, the cytotoxicity correlates with the percentage of L/LMet-MAC released from DSPC/EPG and gradient DSPC liposomes, which were about 50% and 10%, respectively, after 24 h incubation in serum (Figure 3A).

### 3.4. Animal Studies of DSPC/EPG Liposomes and Gradient DSPC Liposome-Encapsulated L/LMet-MAC

#### 3.4.1. Pharmacokinetic Properties in BALB/c Mice

**Figure 5:**
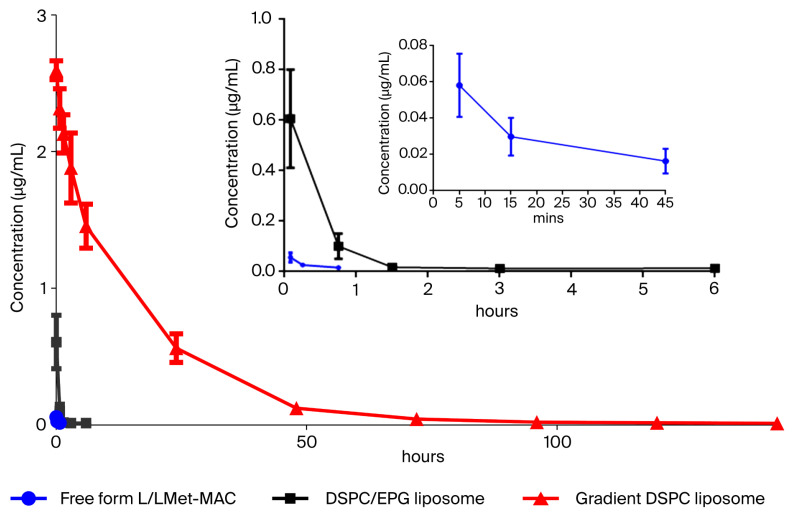
Plasma concentration/time profile of free-form L/LMet-MAC, DSPC/EPG liposome, and gradient DSPC liposome in female BALB/c mice. After a single dose of 5 mg/kg of free-form or liposomal L/LMet-MAC was administrated intravenously into mice, L/LMet-MAC concentration in plasma was analyzed at the indicated time point. Data are presented as mean ± standard deviation (*n* = 5).

Although the in vivo therapeutic efficacy of L/LMet-MAC has been examined [12], its pharmacokinetic (PK) properties have not yet been addressed. To this end, a non-compartment model was used for PK analysis. In the non-compartment model, the AUC (area under the curve) and AUMC (area under the moment curve) are mainly used for calculating pharmacokinetic parameters such as clearance (CL), volume of distribution (Vd), drug mean residence time (mean residual time, MRT), and half-life (t_1/2_) [33]. Table 2 shows the values of these pharmacokinetic parameters of free-form and liposomal L/LMet-MAC. The half-life of free-form L/LMet-MAC was about 23.89 min (Table 2), indicating its poor pharmacokinetic properties and low bioavailability. In contrast, as shown in Table 2 and Figure 5, the half-life of DSPC/EPG liposome and gradient DSPC liposome was approximately about 3.3-fold and 43-fold higher than that of free-form L/LMet-MAC, respectively. Furthermore, the pharmacokinetic parameters of L/LMet-MAC-loaded gradient DSPC liposome were superior to those of DSPC/EPG liposome, which could be explained by two factors. First, the serum stability shown in Figure 3 indicates that DSPC/EPG liposome is less stable than gradient DSPC liposome. Second, incorporating a highly anionic phospholipid such as EPG may accelerate liposome clearance in vivo, as reported by Gabizon et al. [34].

#### 3.4.2. Therapeutic Efficacy in C26 Tumor-Bearing Mice

**Figure 6:**
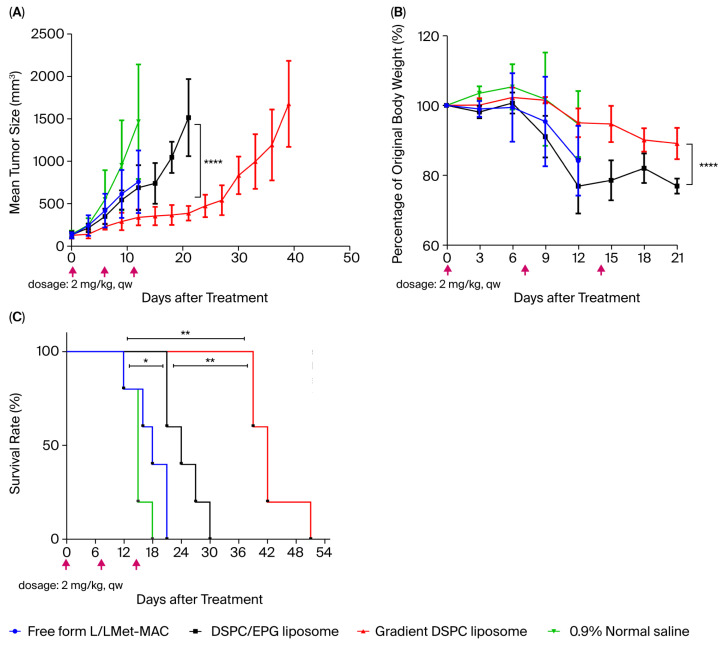
Therapeutic efficacy in mice bearing C26 tumor. Three doses of drug were intravenously injected into the animals of each group (*n* = 5): (**A**) tumor suppressibility profile, (**B**) mouse body weight variation, and (**C**) survival rate of C26-bearing mice. Mice in the respective groups were given free-form or liposomal L/LMet-MAC (2 mg/kg) or 0.9% normal saline via intravenous injection every 7 days for three consecutive weeks. Red arrow heads depict the days of drug injection for the four treatment groups. Tumor size and body weight were measured every 3 days. When the tumor size exceeded 2500 mm^3^, the mice were regarded as dead. The results are presented as the mean ± standard deviation for each group. For panels A and B, statistical analysis was performed using two-way ANOVA over the period from day 0 to day 21. For panel C, statistical differences were analyzed using the log-rank (Mantel–Cox) test. In the two-way ANOVA, **** indicates *p* < 0.0001. In the log-rank test, * indicates *p* < 0.05 and ** indicates *p* < 0.005.

Based on the PK study, we found that gradient DSPC liposome not only could significantly extend the drug half-life but also reduced the volume of distribution (Vd), with a value of 2.40 L/kg. Smith et al. have reported that active pharmaceutical ingredients can be delivered more specifically to the target site if the Vd is less than 5 L/kg [35], implying the possibility of better therapeutic efficacy. To verify this, we further addressed the therapeutic efficacy and toxicity of free-form, DSPC/EPG liposomal and gradient DSPC liposomal L/LMet-MAC in BALB/c mice with C26 tumor. As shown in Figure 6A, compared with the mice receiving 0.9% normal saline, the mean tumor sizes were significantly reduced in the mice that received free-form or DSPC/EPG liposomal L/LMet-MAC. There was no significant difference in tumor reduction between DSPC/EPG liposome and free-form L/LMet-MAC. However, an increase in survival rate was found in the mice receiving DSPC/EPG liposome (Figure 6C), which might relate to the increased half-life of L/LMet-MAC in DSPC/EPG liposome (Figure 5). In fact, the most significant tumor reduction and survival rate were found in the mice treated with gradient DSPC liposomal L/LMet-MAC, which has the longest drug half-life (17.30 h) and smallest Vd (2.40 L/kg). During the PD study, body weight change was used to evaluate adverse effects [36]. As shown in Figure 6B, there was no significant change in body weight in mice given the first dose of DSPC/EPG liposome, gradient DSPC liposome, and 0.9% normal saline. However, the body weight significantly decreased in mice given the second dose of DSPC/EPG liposome or free-form L/LMet-MAC. The body weight loss in mice treated with DSPC/EPG liposome might be caused by drug leakage in the circulation due to the instability of DSPC/EPG liposome in serum (Figure 3). However, the overall toxicity caused by DSPC/EPG liposome is still smaller than that of free-form L/LMet-MAC due to the limited amount of drug that leaked from DSPC/EPG liposome in the circulation. This also explains why the survival rate of mice treated with DSPC/EPG liposome was better than that of mice treated with free-form L/LMet-MAC (Figure 6C).

## 4. Conclusions

In this study, liposomal formulations encapsulated with L/LMet-MAC were developed to improve pharmacokinetics and enhance therapeutic efficacy. We found that PEGylated DSPC liposome prepared with thin-film hydration cannot effectively encapsulate L/LMet-MAC. However, PEGylated DSPC liposomes containing EPG or prepared using the ammonium sulfate gradient method have high encapsulation efficiency (>70%). Compared to DSPC/EPG liposome, gradient DSPC liposome has better stability when incubated with the FBS/PBS mixture. L/LMet-MAC in fact has short blood circulation times (t_1/2_ = 23.89 min), due to rapid excretion by the kidneys. By encapsulating L/LMet-MAC into gradient DSPC liposome, the drug has a long half-life (t_1/2_ = 17.3 h) and displays the lowest volume of distribution in blood circulation that can be released over a prolonged period of time, thereby increasing the bioavailability of the agent and reducing the frequency of dosing. Meanwhile, the particle size of gradient DSPC liposome was around 115 nm, which can be passively targeted to the tumor site via the enhanced permeability and retention (EPR) effect [37]. In fact, L/LMet-MAC-loaded gradient DSPC liposomes show significant therapeutic efficacy and the lowest toxicity in C26-bearing mice. Overall, this study shows that a long-circulating PEGylated DSPC liposome loaded with L/LMet-MAC using the ammonium sulfate gradient approach has favorable pharmacokinetics and an enhanced tumor regressing profile.

## Figures and Tables

**Table 1:** Lipid compositions and characterizations of different types of PEGylated liposomal L/LMet-MAC.

Types	Molar Ratio of Lipid Composition (DSPC/EPG/Chol/DSPE-PEG 2000)	Loading Dose (mg/mL)	Encapsulation Efficiency (%)	Particle Size (nm)	PDI	Lipid Recovery (%)	Drug-to-Lipid Ratio (*w*/*w*)
Conventional DSPC liposome	10:0:5:0.2	0.5	20.28 ± 0.17	104.0 ± 3.7	0.090 ± 0.034	94.88 ± 3.46	0.013 ± 0.001
DSPC/EPG liposome	9:1:5:0.2	0.5	25.39 ± 0.68	115.0 ± 2.3	0.090 ± 0.053	94.55 ± 1.64	0.016 ± 0.001
	5:5:5:0.2	0.5	77.37 ± 1.46	115.4 ± 1.2	0.063 ± 0.021	87.16 ± 2.94	0.056 ± 0.001
	5:5:5:0.2 *	1.0	68.31 ± 5.68	132.1 ± 3.0	0.072 ± 0.022	79.25 ± 4.26	0.109 ± 0.004
Gradient DSPC liposome	10:0:5:0.2	0.5	98.70 ± 0.65	111.4 ± 1.5	0.071 ± 0.043	84.45 ± 3.18	0.074 ± 0.001
	10:0:5:0.2 *	1.0	89.89 ± 2.25	115.6 ± 2.4	0.077 ± 0.040	73.45 ± 1.94	0.155 ± 0.002

Each value represents the mean ± standard deviation from three independent experiments. * Formulations chosen for the stability, in vitro, and in vivo studies.

**Table 2:** Pharmacokinetic parameters of free-form L/LMet-MAC, DSPC/EPG liposome, and gradient DSPC liposome after one intravenous dose of 5 mg/kg in mice.

	Free-Form L/LMet MAC	DSPC/EPG Liposome	Gradient DSPC Liposome
t_1/2_	23.89 min	1.32 h	17.30 h
AUC _0-inf_	2.05 μg·min/mL	0.40 μg·h/mL	43.91 μg·h/mL
AUMC _0-inf_	66.97 μg·min^2^/mL	0.50 μg·h^2^/mL	926.79 μg·h^2^/mL
MRT _0-inf_	32.74 min	1.24 h	21.10 h
Cl	2.44 L/kg/min	12.42 L/kg/h	0.11 L/kg/h
Vd_ss_	80.04 L/kg	15.36 L/kg	2.40 L/kg

t_1/2_ is the half-life of drugs; AUC _0-inf_ is the area under the curve from time zero extrapolated to infinite time; AUMC _0-inf_ is the area under the moment curve from time zero extrapolated to infinite time; MRT _0-inf_ is the mean residence time from time zero extrapolated to infinite time; Cl indicates clearance; Vd_ss_ is the volume of distribution at the steady state. Each value represents an average calculated with PKsolver (*n* = 5).

## Data Availability

Data are available from the corresponding author upon reasonable request.
